# Microstructure and Oxidation Behavior of Fe-25Mn-9Al-8Ni-1C-xTi Alloy Prepared by Vacuum Arc Melting

**DOI:** 10.3390/ma14247722

**Published:** 2021-12-14

**Authors:** Yaping Bai, Keke Tian, Jianping Li, Zhong Yang

**Affiliations:** School of Materials and Chemical Engineering, Xi’an Technological University, Xi’an 710021, China; xiaohua2629@163.com (K.T.); nianbai1116@163.com (J.L.); yz2020611@163.com (Z.Y.)

**Keywords:** Fe-25Mn-9Al-8Ni-1C-xTi, vacuum arc melting, microstructure, oxidation behavior

## Abstract

In this study, Fe-25Mn-9Al-8Ni-1C-xTi alloy (x = 0, 0.1, 0.2, 0.3, 0.4 wt.%) was prepared by vacuum arc melting, and the corresponding microstructure and oxidation behavior at 600 °C were studied. The results show that Fe-25Mn-9Al-8Ni-1C-xTi alloy mainly contains austenite phase, ferrite phase and TiC phase. With Ti content increasing, the austenite phase content decreases, while the contents of ferrite phase and TiC phase increase. The oxidation performance test results show that the addition of Ti element greatly reduces the oxidation weight gain of the alloys at the initial oxidation stage. With the extension of the oxidation time and the further increase of the Ti content, the alloys oxidation weight gain shows a trend of first increasing and then decreasing. When the Ti content is 0.2 wt.%, the oxidation weight gain of this series of alloy reaches the lowest value during the stable oxidation period. Compared with Fe-25Mn-9Al-8Ni-1C alloy, its weight gain per unit area is reduced by 21.1%. Fe-25Mn-9Al-8Ni-1C-xTi alloy oxide layer exhibits a double-layer structure. The outer oxygen layer is mainly loose iron-oxides, while in the inner oxygen layer, the oxides are mainly composed of manganese-oxides and aluminum-oxides, which are relatively dense.

## 1. Introduction

High power density diesel engines are widely used in heavy trucks, tanks and other vehicles because of their excellent fuel economy and power [1,2]. During the operation process, the diesel engine not only bears high temperature and high pressure, but is also subject to high cyclic thermal stress, so the requirements for engine cylinder materials are extremely strict [3,4,5]. The lightweight cylinder block material with excellent mechanical properties can not only reduce the weight of the engine, but also ensure the use safety.

Fe–Mn–Al–C alloy not only shows excellent mechanical properties [6,7,8], but also presents lower density [9,10,11]. Furthermore, with high toughness and strength [12,13,14], good fatigue performance at room temperature [15,16,17] and excellent high temperature oxidation resistance [18,19,20], Fe–Mn–Al–C alloy has great potential in military, industrial and other fields [21,22,23]. Kim et al. studied the effect of intermetallic compounds on Fe-Mn–Al–C alloy and developed the Fe-10Al-15Mn-5Ni-0.8C low-density alloy steel, and they found that the particles with B2 structure could be deposited in the face-centered cubic matrix through appropriate heat treatment process, which greatly improved the mechanical properties and elongation of the alloy [24]. Rahnama et al. found that the microstructure of Fe-15Mn-10Al-5Ni-0.8C alloy was mainly austenite phase after annealing at 1050 °C, and the nano-sized B2 precipitates existed in austenite and composite ferrite [25]. In order to further improve the mechanical properties of Fe–Mn–Al–C alloy, some researchers added a small amount of trace element Ti to the system and found that Ti element can effectively refine the grains [26,27]. Han et al. found that the tempering temperature could reduce the TiC grain size in low-carbon medium manganese steel, and TiC precipitation could effectively refine austenite grains, lead to dislocation strengthening and further improve its strength and toughness [28]. Sarkra et al. found that adding 0.03% Ti can make Fe–Mn–Al–Si–C steel exist good strength and plastic bonding, which was attributed to the precipitation of Ti-rich intermetallic carbides [29]. In the early stage, our research group have researched Fe-25Mn-xAl-8Ni-1C alloy and found that when x = 9, the alloy not only has low density, but also has relatively excellent mechanical properties [30,31].

In addition, researchers have paid more attention to the high temperature oxidation resistance of Fe–Mn–Al–C alloy [18,19,20,32,33]. Barella et al. studied the thermal oxidation resistance of Fe-15Mn-9.5Al-6.5Ni-1Cr-0.43C alloy and found that the alloy structure had a great influence on the oxidation resistance. This is mainly due to the formation of a large amount of σ phase in high temperature conditions, and σ phase and high manganese concentration can promote the oxidation of the alloy [32]. Peng et al. investigated the oxidation behavior of Fe-20Mn-8Al-xC (x = 0.25–0.35 wt.%) duplex light-weight steels at 1000 °C. The corresponding results showed that the increased carbon content enlarged austenite phase fractions and degrades oxidation resistance to high temperature. The preferential oxidation of manganese at high temperature promoted the phase transformation of austenite → ferrite [33].

Therefore, in this research, Fe-25Mn-9Al-8Ni-1C was selected as the matrix alloy. Fe-25Mn-9Al-8Ni-1C-xTi alloy with different Ti contents (0 wt.%, 0.1 wt.%, 0.2 wt.%, 0.3 wt.%, 0.4 wt.%) was prepared by vacuum arc melting. The microstructure and oxidation properties at 600 °C were systematically studied, which could provide theoretical guidance for the development of new-type high-strength and low-density cylinder head materials.

## 2. Preparation and Test Method

### Preparation of Fe-25Mn-9Al-8Ni-1C-xTi Alloy

The raw materials used in this research are industrial pure iron (purity ≥ 99.99), 50% iron-manganese alloy (purity ≥ 99.5), high-purity aluminum particles (purity ≥ 99.99), and high-purity nickel particles (purity ≥ 99.99), Fe-5C alloy (purity ≥ 99.5) and high-purity titanium particles (purity ≥ 99.99). The specific composition ratio is shown in Table 1. The total weight of raw materials for each melting is 100 g. The non-consumable vacuum arc melting equipment produced by Shenyang Jinyan New Material Preparation Technology Co, Ltd. was used for melting. Before melting, a mechanical pump was used to pump a low vacuum, and then a molecular pump was used to pump a high vacuum. When the vacuum degree was 3.0 × 10^−3^ Pa, and the argon gas was started to be filled. Before melting the alloy, high-purity Ti which was put in a copper crucible in the furnace was melted first, and then the iron-based alloys began to be melted in other water-cooled copper crucibles. During the melting process, the arc gun current was gradually increased from the minimum 50 A to 450 A. At the same time, the electromagnetic stirring switch was turned on and each ingot was repeatedly melted four times. After the melting was over, waited for about 30 min and then took out the round cake-shaped alloy ingot. Then the cake-shaped ingot was melted again to form a plate-shaped sample of about 70 × 20 × 8 mm. All melting procedures hold are in one and the same vacuum cycle.

The phases of Fe-25Mn-9Al-8Ni-1C-xTi alloy were analyzed by Bruker D2 PHASER Gen2 X-ray diffraction analyzer (XRD, the scanning speed was 4°/min, the range was 20°–90°, the step length was 0.02). The microstructure and element distribution were analyzed by VEGA II XMU scanning electron microscope (SEM, Quanta-400F), energy spectrometer (EDS, INCA), and field emission high resolution transmission electron microscope (TEM, Talos F200X). The high-temperature oxidation performance of the alloy was tested using the RJ2-15-6 box-type resistance furnace, and the size of the oxidation sample was 8 × 8 × 3 mm. The six faces of each oxidized sample were polished to a mirror finish. The oxidation experiment was performed in a box-type resistance furnace set at 600 °C. After the Al_2_O_3_ ceramic crucible was constant in weight, the oxidized samples were put into the crucible. The samples were taken out and weighed every 10 h to obtain the oxidation curve of mass increasing with time. The oxidation samples were tested by confocal Raman microscope (LabRAM HR Evolution) laser wavelength of 785 nm, scanning range of 100–3200 cm^−1^, combined with XRD test results to determine the surface composition of the oxidized samples. The morphology of the surface layer was observed using SEM. The oxidized sample was cold mounted, polished, and the longitudinal section morphology of the oxidized sample was observed by SEM. Combined with the SEM results of the oxidized surface, the oxidation mechanism was further analyzed.

## 3. Results and Discussion

### 3.1. Phase Diagram Analysis

**Figure 1:**
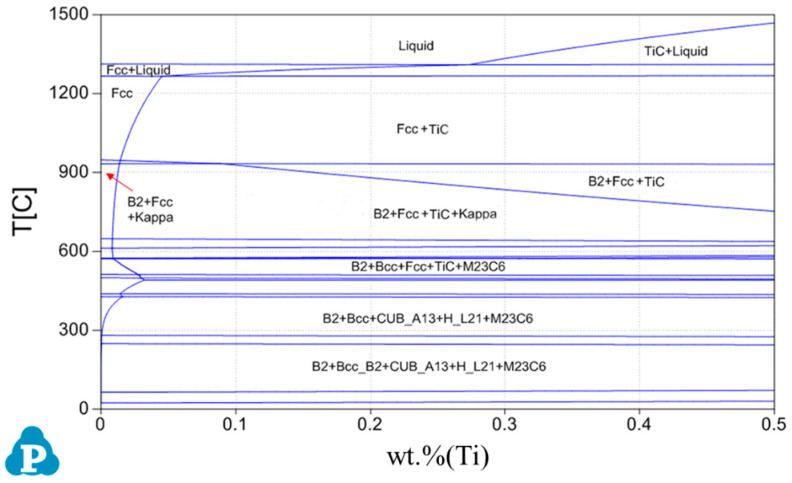
Phase diagram calculation results of Fe-25Mn-9Al-8Ni-1C-xTi alloy.

Pandat software (designed by CompuTherm Company, Middleton, WT, America) is used to calculate the phase diagram of Fe-25Mn-9Al-8Ni-1C-xTi alloy (x = 0 wt.%, 0.1 wt.%, 0.2 wt.%, 0.3 wt.%, 0.4 wt.%, 0.5 wt.%) at room temperature ~1600 °C, as shown in Figure 1. This series of alloys are mainly composed of Fcc phase, Bcc phase, B2 phase, TiC, and k-carbides. One of the Fe-rich Fcc phases is the austenite phase, and the other Fcc phase is the TiC phase. The B2 phase is the ordered phase of the body-centered cubic (Bcc) structure, mainly formed by the NiAl phase. The main Fe-rich phase with much Al content is the ferrite phase.

### 3.2. Microstructure Analysis

**Figure 2:**
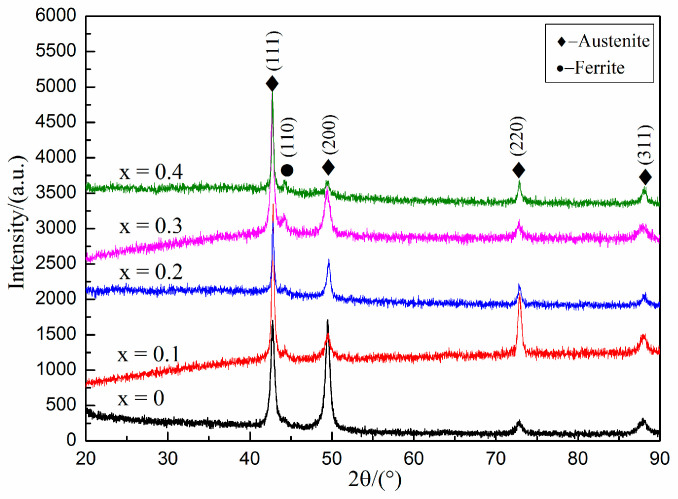
XRD diffraction pattern of Fe-25Mn-9Al-8Ni-C-xTi alloy.

The phase analysis results of Fe-25Mn-9Al-8Ni-1C-xTi alloy are shown in Figure 2. The alloy mainly contains austenite and ferrite phases. Among them, 43.1°, 49.7°, 73.2°, 88.4° are the diffraction peaks of austenite phase (JCPDS: 17-0333), and the intensity of the austenite diffraction peaks gradually weakens with the increase of Ti content. At 44.5°, it is the ferrite diffraction peaks (JCPDS: 06-0696). With the increase of Ti content, the diffraction peak intensity of the ferrite phase gradually increases. The intensity of the diffraction peak at 43.1° will gradually increase with the increase of Ti content.

**Figure 3:**
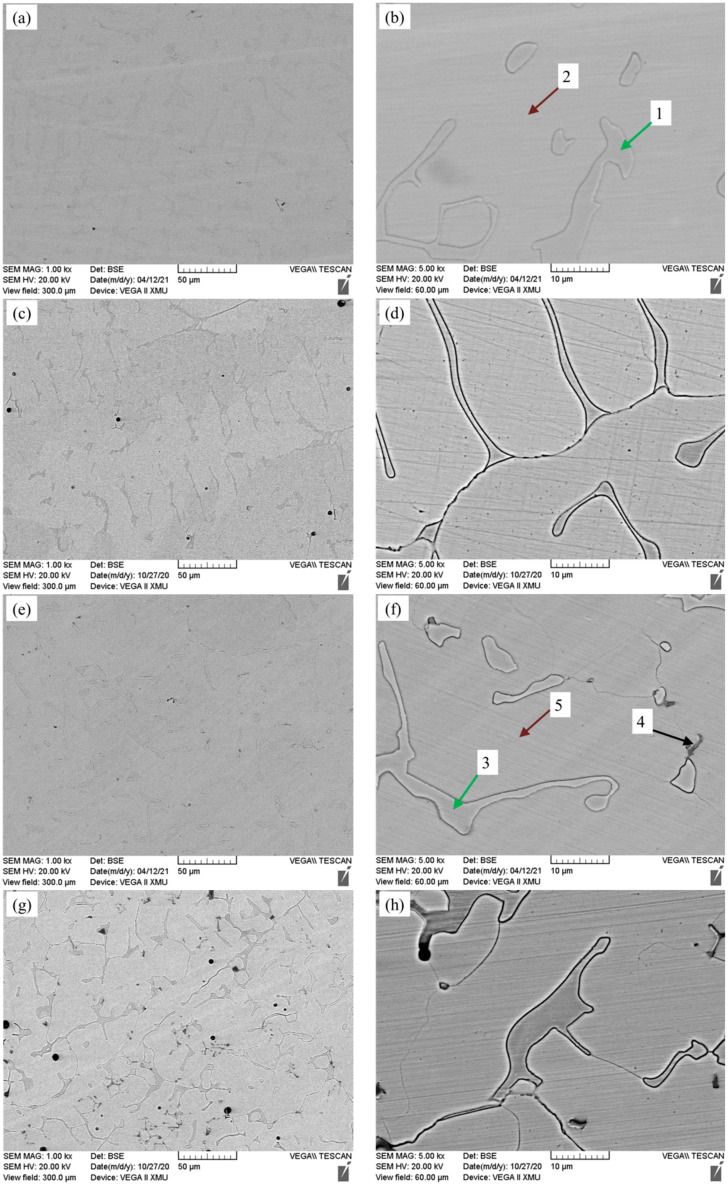

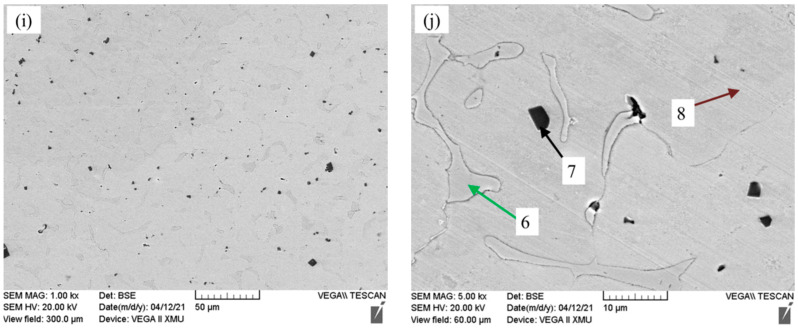
Microstructure of Fe-25Mn-9Al-8Ni-1C-xTi alloy. (**a**,**b**) 0 wt.% Ti; (**c**,**d**) 0.1 wt.% Ti; (**e**,**f**) 0.2 wt.% Ti; (**g**,**h**) 0.3 wt.% Ti; (**i**,**j**) 0.4 wt.% Ti.

The microstructure of the Fe-25Mn-9Al-8Ni-1C-xTi alloy is present in Figure 3, and the corresponding EDS analysis results (at.%) in regions 1–8 are presented in Figure 3b,f,g and summarized in Table 2. From Figure 3a and the corresponding enlarged Figure 3b, it can be seen that there are mainly two phases in Figure 3a, one of which is the matrix phase and the other is a continuously distributed network phase. However, when the Ti content further increased to 0.3% (Figure 3g,h), a small amount of darker areas phase appeared in the microstructure. Combined with the results of EDS analysis in Table 2, the content of C and Mn in point 2, point 5 and point 8 is relatively higher than that in point 1, point 3 and point 6, and the content of Ni and Al is relatively lower than that in point 1, point 3 and point 6. Based on this, it is inferred that the point 2, point 5 and point 8 correspond to the austenite phase, and the point 1, point 3 and point 6 correspond to the ferrite phase. It can be seen from Figure 3 that with the increase of Ti content, the content of darker areas in the alloy microstructure also increases. Due to the content of Ti and C in point 4 is large, and the content of C is almost twice as Ti, it is inferred that this point is Ti based carbide. From the point analysis of darker areas phase shown as point 7, this area is mainly enriched by Ti and C, and the atomic percentage of Ti and C is close to 1:1. It is deduced that it is TiC phase.

**Figure 4:**
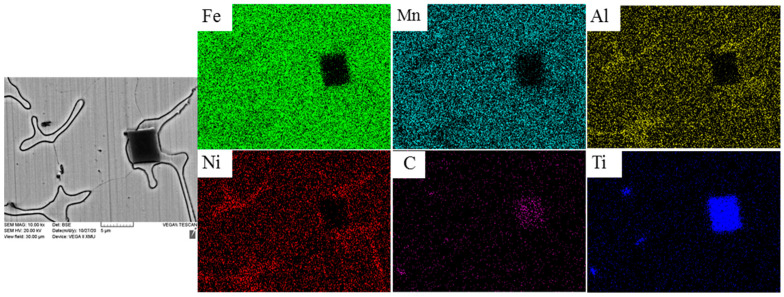
SEM image and element distribution of Fe-25Mn-9Al-8Ni-1C-0.4Ti alloy.

The SEM image of Fe-25Mn-9Al-8Ni-1C-0.4Ti alloy and element distribution are present in Figure 4. Where Fe and Mn elements are evenly distributed in this area, Ni and Al elements combine to form network phase. It is speculated that the network phase is ferrite and is widely distributed on the austenite matrix [34,35]. While in the square area, Ti element and C element are enriched, so it can be inferred that this area is titanium carbon compound.

**Figure 5:**
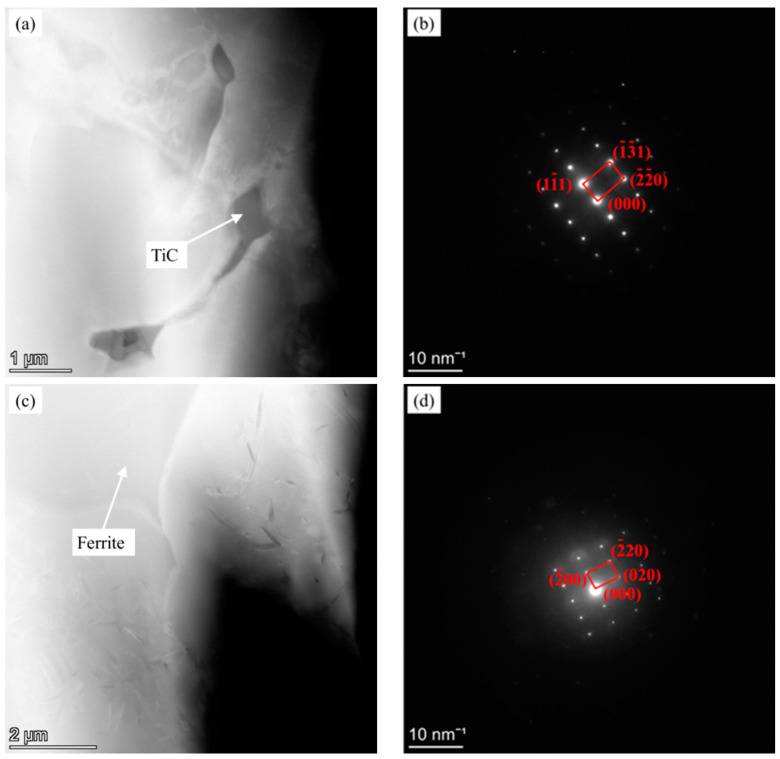
TEM image of Fe-25Mn-9Al-8Ni-1C-0.4Ti alloy. (**a**) BF TEM of TiC; (**b**) TiC diffraction spots (**c**) BF TEM of Ferrite; (**d**) Ferrite diffraction spots.

To further judge the type of titanium carbon compound in the microstructure, Fe-25Mn-9Al-8Ni-1C-0.4Ti alloy is analyzed by TEM. The corresponding Bright Field Transmission Electron Microscopy (BF TEM) micrograph is shown in Figure 5a and the corresponding diffraction spots are shown in Figure 5b. According to the analysis of diffraction patterns (JCPDS: 32-1383), it can be further determined that the addition of Ti does produce TiC phase in the matrix alloy. In order to further investigate whether the continuously distributed network phase is ferrite phase in the alloy, TEM analysis was performed on it. The corresponding BF TEM micrograph is shown in Figure 5c and the corresponding diffraction spots are shown in Figure 5d. According to the analysis of diffraction patterns (JCPDS: 6-0696), it is further determined that it is ferrite phase.

### 3.3. Oxidation Resistance Analysis

**Figure 6:**
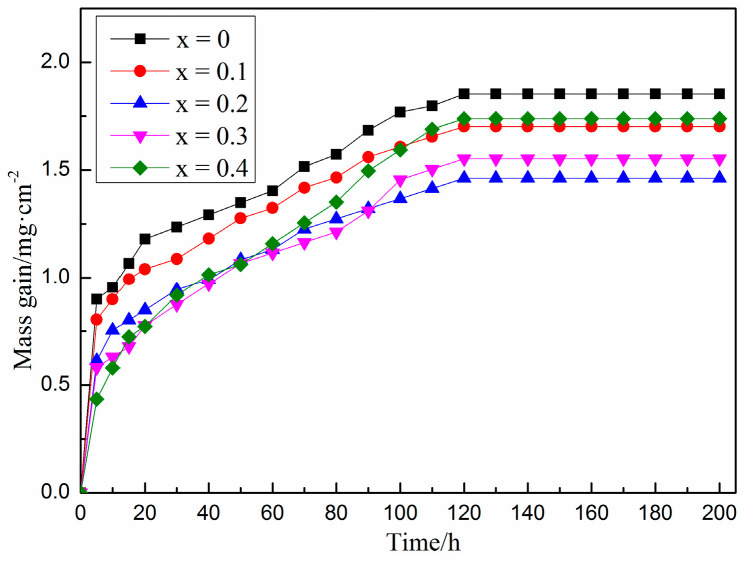
Fe-25Mn-9Al-8Ni-1C-xTi alloy oxidation weight gain curve.

The weight gain curve of Fe-25Mn-9Al-8Ni-1C-xTi alloy after holding at 600 °C for 200 h is present in Figure 6. It can be concluded from Figure 6 that under the same conditions, the oxidative weight gain of the alloy shows a trend of first decreasing and then increasing with the increase of Ti content. Among them, the 0.2 wt.% Ti alloy has the smallest oxidative weight gain. During the first 20 h of oxidation, the more Ti content, the more excellent oxidation resistance of the alloy, and the oxidative weight gain of the alloy without Ti element is 1.179 mg/cm^2^, while 0.2 wt.% Ti alloy is as low as 0.848 mg/cm^2^ and 0.4 wt.% Ti alloy is only 0.773 mg/cm^2^. With the extension of time, the oxidation weight gain of this series of alloys remained stable at 120 h, and the oxidation weight gain of alloy with 0 wt.%, 0.1 wt.%, 0.2 wt.%, 0.3 wt.%, 0.4 wt.% Ti content is 1.854 mg/cm^2^, 1.703 mg/cm^2^, 1.462 mg/cm^2^, and 1.553 mg/cm^2^, 1.738 mg/cm^2^, respectively. It can be judged that with the addition of Ti content, the oxidation resistance of this series of alloy shows a trend of first strengthening and then weakening. Compared with the alloy without Ti, the oxidation weight per unit area of 0.2 wt.%Ti alloy is reduced by 21.1%. Compared with 0.2 wt.%Ti alloy, 0.4 wt.%Ti alloy weight gain per unit area increased by 18.8%. It is speculated that Ti as an active element, the addition of right amount can reduce the oxidation rate of the alloy in high temperature environment. It can be seen from Figure 2 that with the increase of Ti content, the austenite phase content in the alloy gradually decreases, and the ferrite content gradually increases. The content of aluminum is higher in ferrite, and the content of manganese is higher in austenite. The oxide of austenite is mainly composed of manganese oxide, while the oxide of ferrite is mainly alumina in high temperature conditions. Therefore, ferrite is more stable in high temperature conditions and has better oxidation resistance [36]. However, with the increase of TiC content, the grain boundaries increase in the alloy. The binding force and thermal expansion coefficient of TiC are different with austenite and ferrite. Therefore, the O element easily diffuses inward from the interface between TiC, austenite and ferrite, which weakens the oxidation resistance of the alloy in high temperature conditions.

**Figure 7:**
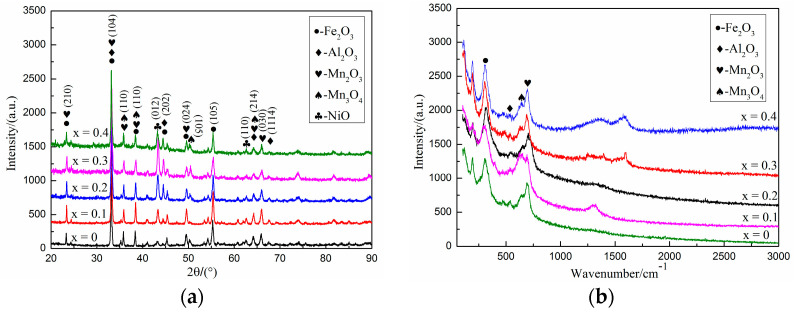
XRD pattern and Raman pattern of the surface layer of Fe-25Mn-9Al-8Ni-1C-xTi alloy oxidized for 200 h. (**a**) XRD pattern; (**b**) Raman pattern.

XRD tests were carried out on the surface of the alloys with different Ti content after oxidation, and the results are present in Figure 7a. As can be seen from Figure 7a, after oxidation of Fe-25Mn-9Al-8Ni-1C-xTi alloy at 600 °C, the series of oxide layers mainly contain oxides such as Fe_2_O_3_ (JCPDS: 33-0664), Al_2_O_3_ (JCPDS: 51-0769), and Mn_2_O_3_ (JCPDS: 41-1442). It can also be inferred from Figure 7a that there is no Fe^2+^ in the oxidized surface layer of the alloy, which is mainly due to the fact that during the oxidation process, Fe is first oxidized to divalent Fe ions to form FeO. Because Fe^2+^ is unstable, it will be further oxidized to form Fe_3_O_4_. However, the two Fe ions are +3 valence, and one is +2 valence in Fe_3_O_4_, so the oxide is still unstable [37]. Divalent Fe ions will be further oxidized, and eventually all Fe ions will be oxidized to Fe^3+^, forming stable Fe_2_O_3_. In addition, a small amount of Mn_3_O_4_ (JCPDS: 18-0803) and Al_2_O_3_ were found from XRD. This is because at the initial stage of oxide film formation, Al and Mn element can fully contact with O element to form oxide film. Al element in the ferrite easily diffuses from the grain boundary to the surface layer, forming a dense oxide film, which prevents its further oxidation. Raman spectroscopy was performed on the surface of the samples after oxidation of alloys with different Ti content, and the test results are present in Figure 7b. It can be seen from Figure 7b that the oxide on the surface of Fe-25Mn-9Al-8Ni-1C-xTi alloy is mainly composed of Fe_2_O_3_, Al_2_O_3_, Mn_2_O_3_ and Mn_3_O_4_, which is consistent with the XRD test results (as shown in Figure 7a).

**Figure 8:**
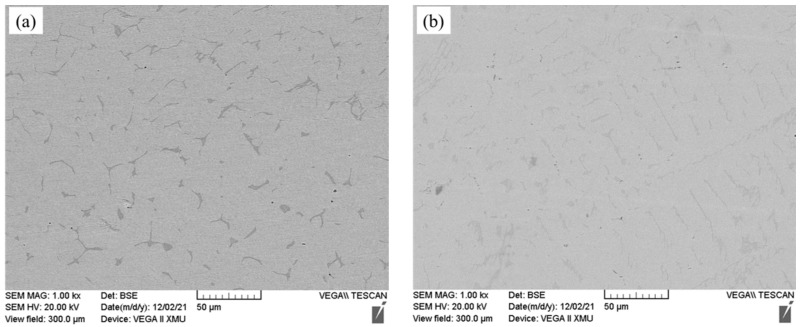

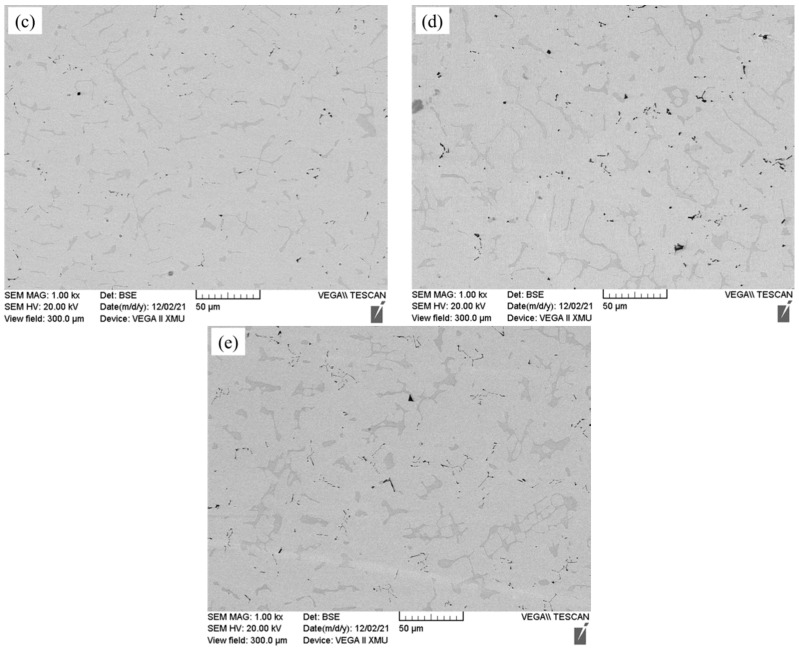
The micrograph for the surface morphology of Fe-25Mn-9Al-8Ni-1C-xTi alloy before the oxidation. (**a**) 0 wt.% Ti; (**b**) 0.1 wt.% Ti; (**c**) 0.2 wt.% Ti; (**d**) 0.3 wt.% Ti; (**e**) 0.4 wt.% Ti.

**Figure 9:**
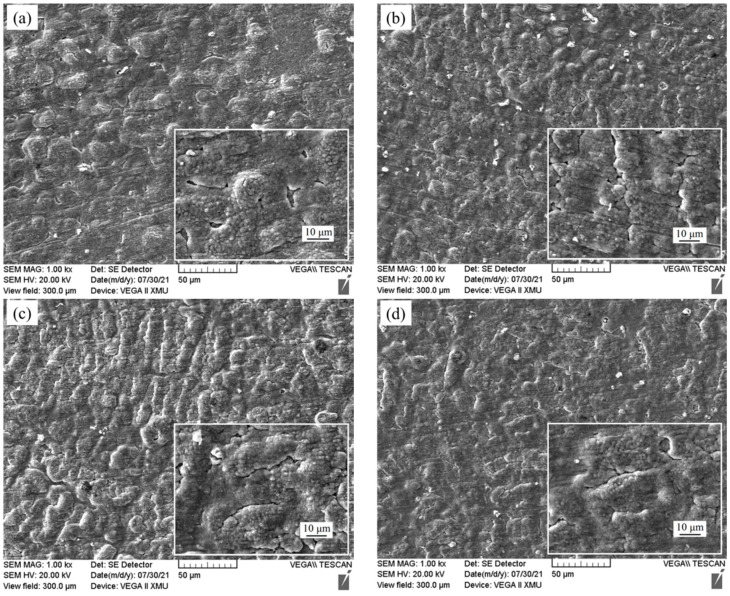

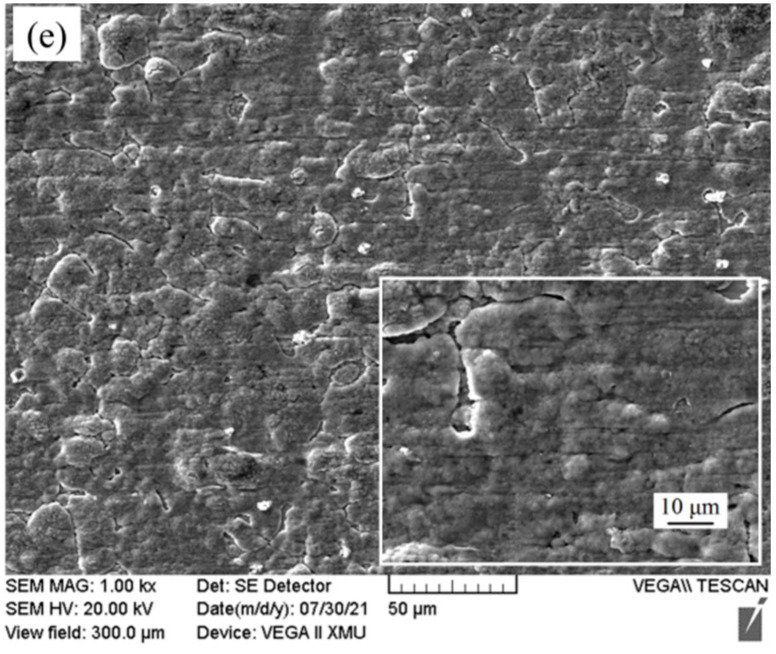
Surface morphology of Fe-25Mn-9Al-8Ni-1C-xTi alloy after 30 h oxidation. (**a**) 0 wt.% Ti; (**b**) 0.1 wt.% Ti; (**c**) 0.2 wt.% Ti; (**d**) 0.3 wt.% Ti; (**e**) 0.4 wt.% Ti.

**Figure 10:**
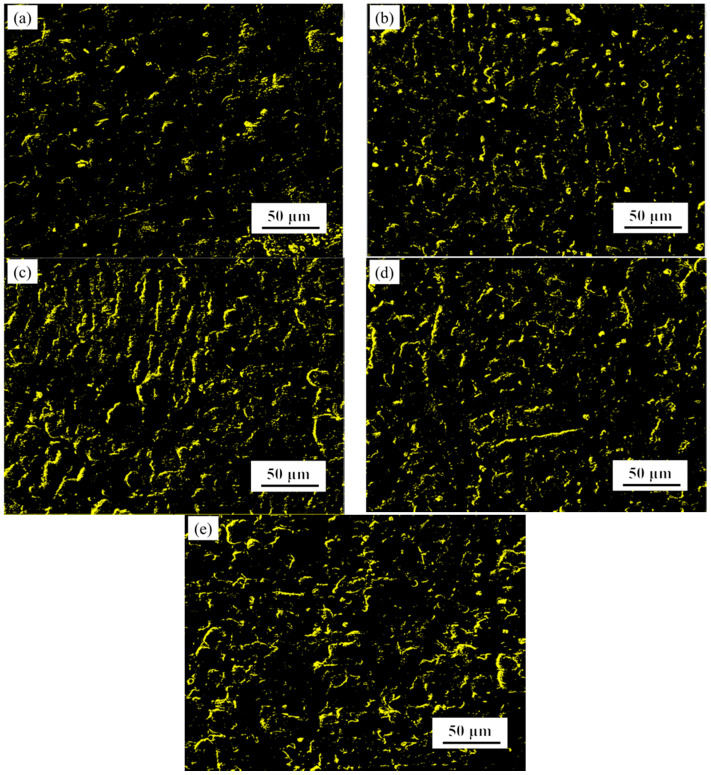
The granular oxide distribution on the oxidization surface of Fe-25Mn-9Al-8Ni-1C-xTi alloy. (**a**) 0 wt.% Ti; (**b**) 0.1 wt.% Ti; (**c**) 0.2 wt.% Ti; (**d**) 0.3 wt.% Ti; (**e**) 0.4 wt.% Ti.

In order to further study the oxidation mechanism of the Fe-25Mn-9Al-8Ni-1C-xTi alloy, samples were taken after 30, and 200 h to observe the morphology of the oxidation surface. In order to clearly compare the changes of the surface of Fe-25Mn-9Al-8Ni-1C-xTi alloy before and after oxidation, the surface of this series of alloys before oxidation was observed by SEM, and the results are shown in Figure 8. The surface morphology of Fe-25Mn-9Al-8Ni-1C-xTi alloy oxidized for 30 h is present in Figure 9. The morphology of the alloy oxidation surface has more gray-back cellular protrusions. Some bright white substance fine granular oxides appeared on the cellular protrusions. Many fine granular oxides appeared on the cellular protrusions. It is caused by the further growth of the surface oxide. Compared with the alloy surface oxide without Ti element and the alloy with Ti element added, the alloy with Ti element oxidized less cellular protrusions and the protrusions are flatter. Figure 10 shows the granular oxide distribution on the oxidization surface of Fe-25Mn-9Al-8Ni-1C-xTi alloy. The granular oxides are colored in yellow. The proportion of granular oxides of Fe-25Mn-9Al-8Ni-1C-xTi alloy are also counted by image processing software, and the statistical results are 14.45% (0 wt.% Ti), 15.02% (0.1 wt.% Ti), 19.29% (0.2 wt.% Ti), 17.96% (0.3 wt.% Ti) and 16.57% (0.4 wt.% Ti), respectively. Among them, the amount of granular oxide on the alloy surface added with 0.2 wt.% Ti is the largest. It indicates that 0.2 wt.% Ti element can promote more formation Al_2_O_3_ on the surface, which can effectively prevent further oxidation of the alloy.

**Figure 11:**
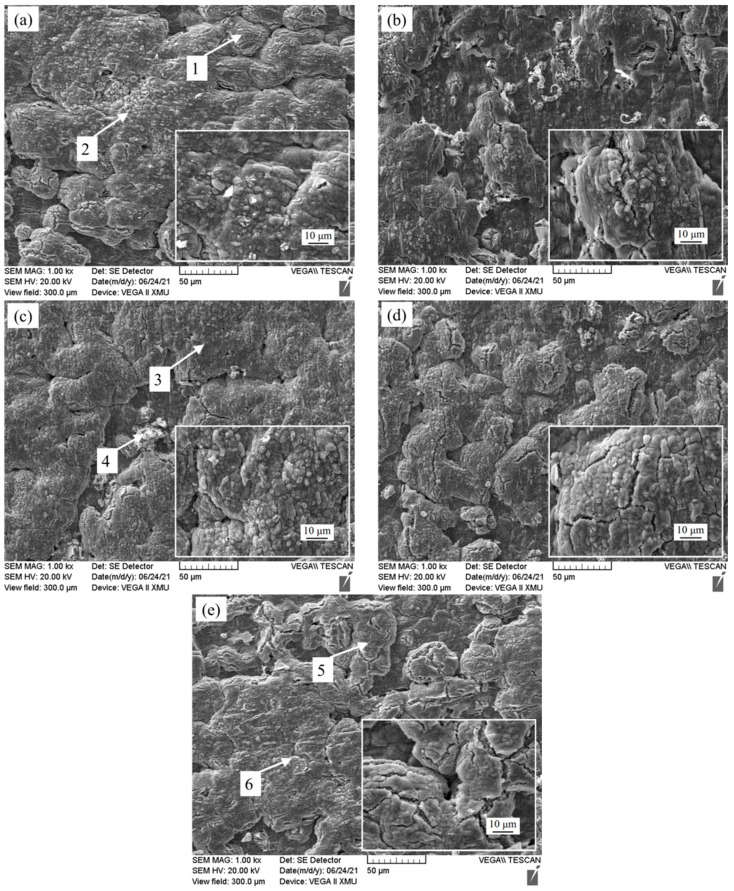
Surface morphology of Fe-25Mn-9Al-8Ni-1C-xTi alloy after 200h oxidation. (**a**) 0 wt.% Ti; (**b**) 0.1 wt.% Ti; (**c**) 0.2 wt.% Ti; (**d**) 0.3 wt.% Ti; (**e**) 0.4 wt.% Ti.

The surface morphology of Fe-25Mn-9Al-8Ni-1C-xTi alloy oxidized for 200 h is present in Figure 11. It can be seen from Figure 11 that the oxide surface layer is mainly composed of two forms of substances. One is a continuously distributed gray-black substance, and the other is a dotted bright white substance attached to the gray black substance. They are a cellular bulge, and the gray-black material is continuously distributed on the surface. With the increase of Ti content, there are less and less bright white substances in the oxidized surface layer, and the surface becomes more and more smooth, showing that gray-black cellular protrusions occupy the oxidized surface layer. The EDS energy spectrum analysis of different positions in Figure 11 is present Table 3. It can be seen from the EDS energy spectrum that the gray-black cellular protrusion of the alloy oxidation surface is mainly composed of O element, Mn element and Fe element, and the content of Mn element is more than that of Fe element. Therefore, it is inferred that the material is composed of Mn oxide and a small amount of Fe oxide. The bright white protrusions contain a large amount of Mn and Fe elements, and it is speculated that this is the result of the simultaneous presence of oxides of Mn and Fe elements. No Ni oxide was found on the surface layer, mainly due to the small diffusion coefficient of Ni [38]. In the process of O diffusion, Fe and Mn element combine with O element before Ni to produce redox reaction, it can be seen from Figure 7 that the oxidation products are Fe_2_O_3_, Mn_2_O_3_, Mn_3_O_4_.

**Figure 12:**
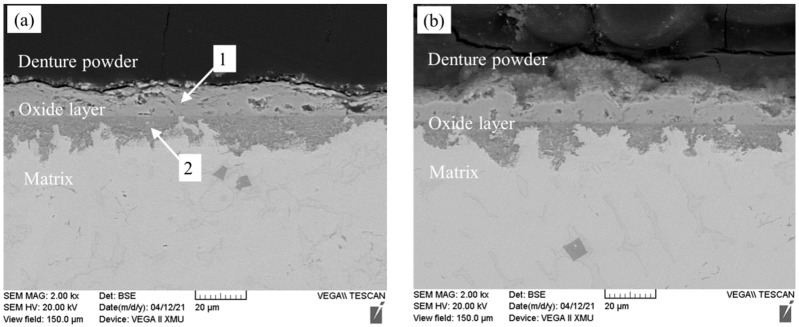

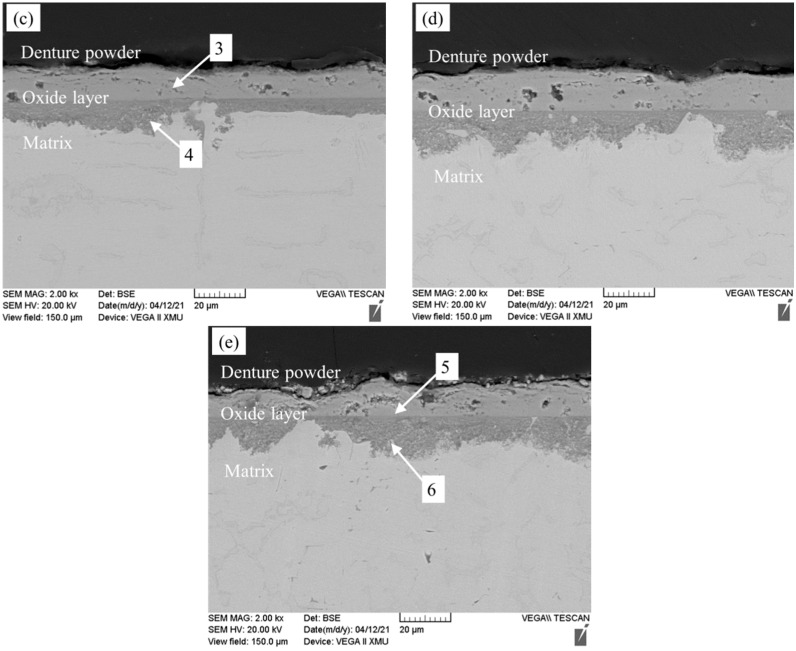
Longitudinal cross-sections of Fe-25Mn-9Al-8Ni-1C-xTi alloy oxidized for 200 h. (**a**) 0 wt.% Ti; (**b**) 0.1 wt.% Ti; (**c**) 0.2 wt.% Ti; (**d**) 0.3 wt.% Ti; (**e**) 0.4 wt.% Ti.

SEM analysis of the longitudinal section of Fe-25Mn-9Al-8Ni-1C-xTi alloy after oxidation is shown in Figure 12. Serving the denture powder on the top surface of the samples, it can prevent the oxide layer from falling off when making the oxide cross-sectional sample. After 200 h of high temperature oxidation, the oxidized layer is divided into two layers (the inner oxidized layer and the outer oxidized layer). There is a clear interface between the inner oxidized layer and the outer oxidized layer. The internal oxidation layer is denser and no holes. The main reason for the two types of oxide layers is that during the process of oxidation and diffusion of O atoms, O atoms will first react with Fe, Mn and Al elements on the surface of the metal, which further weakens the O atoms entering the alloy during the diffusion process. The average thickness of the oxide layer is measured by software Gatan DigitalMicrongraph (designed by AMETEK Company, Pleasanton, CA, USA). Through measurement and calculation, it is listed in the following Table 4. As can be seen from Table 4, when the addition amount of Ti is 0, the oxide layer thickness of the alloy is 29.410 μm, while the oxide layer thickness of the alloy is 25.467 μm when the addition amount of Ti is 0.2 wt.%. The oxidized layer thickness of the 0.2 wt.% Ti alloy is reduced by 3.943 μm (13.5%) compared with the alloy with no Ti addition, and the oxidized layer thickness of 0.4 wt.% Ti alloy is reduced by 0.244 μm (8.3%).

The EDS energy spectrum analysis of the oxidation section of Fe-25Mn-9Al-8Ni-1C-xTi alloy is present in Table 5. According to the EDS analysis at point 1, 3, and 5 in the outer oxidized layer, it can be seen that there are more Fe elements but almost no Al, Ni elements. However, the oxide formed by Fe element has poor compactness and it is easy to form loose pores. While at point 2, 4, and 6, there are much content of Al element, and also a certain amount of Mn, Ni element in the inner oxidized layer. Compared with the outer oxidized layer, Al is the forming element of ferrite. Therefore, during the oxidation of alloy at 600 °C for 200 h, Al element diffuses outward along the grain boundaries of austenite and ferrite, and O diffuses inward along the alloy surface. These two elements form Al_2_O_3_ in the inner oxide layer of the alloy, and the Al_2_O_3_ oxide is relatively dense, which can effectively prevent the further diffusion of O element.

It can also be found that the aluminum content in the inner oxygen layer is obviously higher than that of the outer oxygen layer. This is mainly due to the increase of Ti, the formation of Ti-based carbides increases in the alloy. O element further diffuses into the inner oxidized layer along the interface of the TiC phase, the ferrite phase and the austenite phase. In addition, the content of Ni is lower than that of Fe and Mn, and its outward diffusion rate is slower than that of Fe and Mn, the content of Ni in the inner oxidized layer is more than that of the outer oxidized layer. Ti addition leads to the content increase of ferrite with good oxidation resistance in the matrix. Therefore, after Ti addition, the thickness of the inner oxidized layer is less than that of the material without Ti, and the comprehensive oxidation resistance is improved.

**Figure 13:**
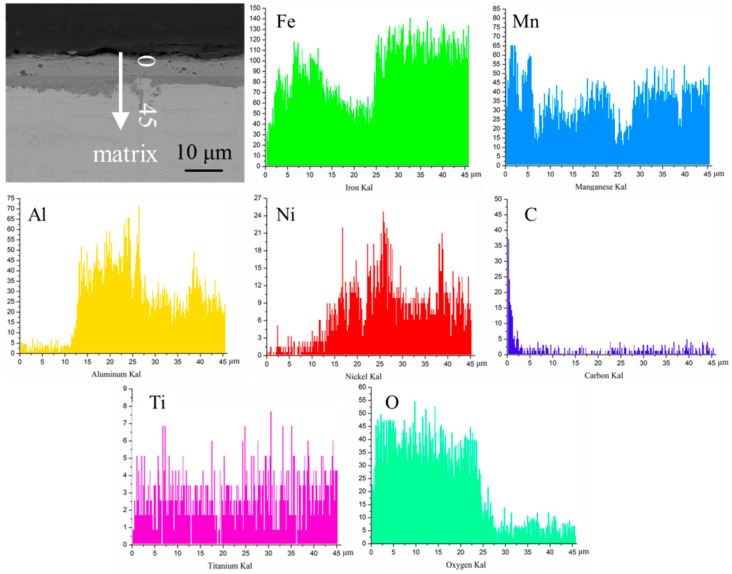
Element line scan of the Fe-25Mn-9Al-8Ni-1C-0.2Ti alloy oxidation longitudinal cross-section.

The line scan analysis of the oxidation longitudinal section of Fe-25Mn-9Al-8Ni-1C-0.2Ti alloy is present in Figure 13. Through its line scan, it can be found more intuitively that the content of Al and Ni is less at the outer oxidized layer, and the content of Al at the inner oxidized layer is significantly higher than that in the not oxidized area. Besides, the content of Mn element in the outer oxidized layer is higher than that in the not oxidized area. It is speculated that the main reason is that Mn element easily diffuses to the surface of the alloy during the oxidation process, thereby generating more oxides. The oxide of Mn is denser than the oxide of Fe, which can prevent further oxidation from occurring. However, the elements of Al and Ni do not appear in the outer oxidized layer, indicating that the migration rate of these two elements during the oxidation process was small, much lower than that of Fe and Mn, which further indicated that the part to prevent oxidation was mainly the inner oxidized layer. It can be concluded from the line scan of the oxidation longitudinal section that the O element only exists in the inner and outer oxidized layers, and not diffuse into the alloy. It indicates that the oxidized layer effectively prevents the further oxidation of the alloy and makes it have a certain degree of oxidation resistance.

## 4. Conclusions

In this study, Fe-25Mn-9Al-8Ni-1C-xTi (Ti content is 0 wt.%, 0.1 wt.%, 0.2 wt.%, 0.3 wt.%, 0.4 wt.%) alloy was prepared by vacuum arc melting. The microstructure and oxidation properties of this series of alloy were studied. The following conclusions were made:

(1) Fe-25Mn-9Al-8Ni-1C-xTi alloy mainly contains austenite, ferrite and a small amount of TiC. As the Ti content increases, the austenite content in the alloy gradually decreases, and the ferrite and TiC content gradually increases.
(2) The oxidation performance test results of Fe-25Mn-9Al-8Ni-1C-xTi at 600 °C present that the addition of Ti element greatly reduces the weight gain of the alloy in the initial stage of oxidation. With the extension of the oxidation time and the further increase of the Ti content, the alloy oxidation weight gain shows a trend of first increasing and then decreasing. When the Ti content is 0.2 wt.%, the oxidation weight gain of the alloy in the stable oxidation period reaches the lowest value. The oxidation weight gain is 1.462 mg/cm^2^, which is 21.1% less than the weight gain per unit area of the alloy without Ti.
(3) The oxide layer of Fe-25Mn-9Al-8Ni-1C-xTi alloy exhibits a double-layer structure. The outer oxidized layer mainly forms Fe oxide, and its structure is loose. The inner oxide layer is mainly the oxide formed by manganese and aluminum, which are Mn_2_O_3_ and Al_2_O_3_ respectively, and they are relatively dense. It can effectively prevent the diffusion of oxygen into the alloy. When the Ti content is 0.2 wt.%, the oxidized layer of the alloy is the thinnest at 25.467 μm, which is 3.943 μm (13.5%) less than the alloy without Ti addition.

## Figures and Tables

**Table 1:** Fe-25Mn-9Al-8Ni-1C-xTi alloy composition ratio scheme (Mass/g).

Alloy	Variable-x	Industrial Pure Iron	50%Fe-Mn Alloy	Aluminum Particles	Nickel Particles	Fe-5C Alloy	Titanium Particles
Fe-25Mn-9Al -8Ni-1C-xTi (0 ≤ x ≤ 0.4)	0	13	50	9	8	20	0
	0.1	12.9	50	9	8	20	0.1
	0.2	12.8	50	9	8	20	0.2
	0.3	12.7	50	9	8	20	0.3
	0.4	12.6	50	9	8	20	0.4

**Table 2:** EDS analysis of Fe-25Mn-9Al-8Ni-1C-xTi alloy in Figure 3.

Point	Element/(at.%)					
	Fe	Mn	Al	Ni	C	Ti
1	43.53	14.54	26.26	15.58	/	0.09
2	44.39	17.42	12.09	5.00	21.08	0.02
3	40.32	13.22	21.82	11.10	13.46	0.08
4	12.36	7.65	2.44	1.78	51.36	24.41
5	45.17	16.22	12.60	4.79	20.98	0.24
6	39.01	12.82	22.91	13.09	12.02	0.15
7	1.73	1.13	0.70	0.33	48.59	47.52
8	41.65	17.74	12.56	5.48	22.57	/

**Table 3:** EDS analysis of the surface layer of Fe-25Mn-9Al-8Ni-1C-xTi alloy oxidized for 200 h.

Point	Element/(at.%)						
	Fe	Mn	Al	Ni	C	Ti	O
1	9.41	30.51	/	0.18	2.24	/	57.66
2	15.58	19.19	/	0.07	5.69	/	59.47
3	11.07	29.56	0.08	0.15	3.54	/	55.60
4	13.62	23.53	/	/	6.33	0.07	56.45
5	8.00	22.68	/	0.11	3.83	0.05	65.51
6	12.68	20.15	/	0.03	7.06	0.02	60.06

**Table 4:** The average thickness of oxidation layer of alloy with 600 °C insulation for 200 h.

Alloy	Variable-x	Average Thickness (μm)
Fe-25Mn-9Al-8Ni-1C-xTi (0 ≤ x ≤ 0.4)	0	29.410
	0.1	28.454
	0.2	25.467
	0.3	26.476
	0.4	29.166

**Table 5:** EDS analysis of longitudinal cross-sections of Fe-25Mn-9Al-8Ni-1C-xTi alloy oxidized for 200 h.

Point	Element/(at.%)						
	Fe	Mn	Al	Ni	C	Ti	O
1	34.75	8.96	0.20	0.10	0.46	/	55.53
2	14.48	7.37	20.26	13.5	0.40	/	43.99
3	36.80	6.02	0.03	0.12	0.53	/	56.50
4	18.91	11.22	15.99	5.30	0.46	0.12	50.00
5	41.31	6.17	0.10	0.42	0.47	/	51.53
6	17.75	10.09	18.50	2.86	0.27	0.14	50.39

## Data Availability

All the data is available within the manuscript.
